# Clinical Outcomes and Risk Factor Analysis of Patients Presenting with Emphysematous Cystitis: A 15-Year Retrospective Multicenter Study

**DOI:** 10.3390/medicina57060531

**Published:** 2021-05-26

**Authors:** Jeonghyouk Choi, Seung-Kwon Choi, Sang-Hyub Lee, Koo-Han Yoo

**Affiliations:** 1 Department of Urology, College of Medicine, Kyung Hee University, Seoul 02447, Korea; jhchoi0@gmail.com (J.C.); uroshlee@khu.ac.kr (S.-H.L.); 2 Department of Urology, Seoul Medical Center, Seoul 02053, Korea; urocsk0127@hanmail.net

**Keywords:** cystitis, emphysema, risk factors, sepsis, mortality

## Abstract

*Background and objectives:* To investigate the risk factors for emphysematous cystitis (EC) compared to those of acute cystitis (AC) to increase clinicians awareness of the possibility for the aggravation of patient status. *Materials and methods:* We retrospectively reviewed a total of 54 patients who were hospitalized with a diagnosis of EC by abdominal computed tomography (CT) scan from 2006 to 2020. The control group included 92 patients who were hospitalized for the treatment of AC in the same period. We sought to identify the clinical features and predisposing diseases, such as age, sex, diabetes mellitus (DM), hypertension (HTN), cerebrovascular accident (CVA), chronic kidney disease (CKD), neurogenic bladder (NB), history of urinary tract infection (UTI), and emphysematous pyelonephritis (EPN), that were associated with the development of EC. *Results:* The median (interquartile range (IQR)) age of the patients with EC was older than that of the patients with AC (78.5 (15.3) years (range: 52–100) vs. 70.0 (26.5) years (range: 28–97 years)). Sepsis and mortality occurred only in the EC group (48.1% and 11.1%, respectively). The univariate analysis of predisposing factors revealed that age, DM, HTN, CVA, CKD, and NB were significantly associated with EC. In the multivariate analysis, DM (OR, 6.251; 95% CI, 2.254–17.250; *p <* 0.001), CKD (OR, 18.439; 95% CI, 3.421–99.404; *p =* 0.001), NB (OR, 7.374; 95% CI, 1.993–27.285; *p =* 0.003) were associated with EC. *Conclusions:* The results of this study revealed that DM, CKD, and NB were significant risk factors for EC. The tendency toward sepsis and high mortality underscore the need for careful observation while treating patients with EC with the risk noted above.

## 1. Introduction

Emphysematous urinary tract infection (UTI), including emphysematous pyelonephritis (EPN) and emphysematous cystitis (EC), is characterized by gas-forming infections in related organs. While most sources of the gas are bacteria, several cases of EC were reportedly induced by chemicals [1]. Among gas-forming infections, EPN easily develops to severe necrosis of the kidney and perirenal tissues with a high mortality rate of 21%. Meanwhile, EC shows infection of the bladder wall and lumen with presentations varying from nonspecific clinical symptoms of cystitis to systemic response, including fever and myalgia up to urosepsis (mortality rate, 7%) [2,3,4].

The first case of pneumaturia, which was most likely a symptom of EC, was reported in 1671 [5]. The incidence of EC is unknown [6]. Eisenlohr first identified emphysema in the bladder by autopsy in the late 1800s. In 1961, Bailey reported the relationship between pneumaturia and EC, and defined EC [7]. Despite the high mortality rates of EC, recent studies on EC are sparse due to its rarity. The risk factors for EC are older women, diabetes mellitus, neurogenic bladder, urinary tract outlet obstruction, recurrent UTI, and urethral catheter placement [8,9,10]. EC is diagnosed based on the presence of air in the bladder wall or lumen on radiologic examinations, including abdominal radiography or computed tomography (CT) [6]. Most patients are treated successfully with antibiotics, urethral catheterization and hydration. However, surgical treatment, such as cystectomy, is required in 10% of patients with EC [7].

While acute cystitis (AC) is a common disease, EC is very rare and can be fatal. Therefore, clinicians must discriminate AC from EC. However, when a clinician encounters a patient with AC in an outpatient clinic, it may be difficult to determine whether to perform imaging tests such as CT or to start aggressive treatment for EC.

## 2. Material and Methods

### 2.1. Study Aim

The aim of our study was to investigate the risk factors for EC compared to those of AC and to help clinicians to prepare for the possibility of the aggravation of patient status.

### 2.2. Study Design

**Figure 1:**
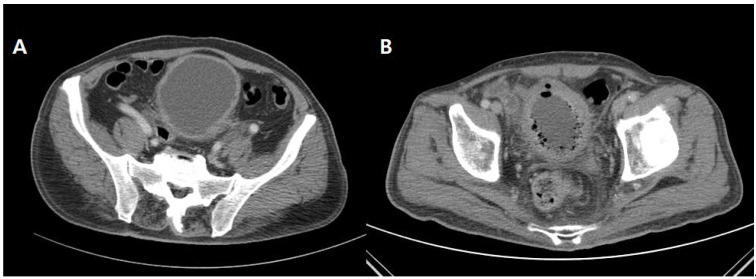
Axial computed tomography (CT) image through the lower pelvis of patients with acute cystitis (**A**) and emphysematous cystitis (**B**), with diffuse bladder wall thickening shown in the bladder of the patient with acute cystitis and gas collection within the bladder wall and lumen in the patient with emphysematous cystitis.

We retrospectively reviewed patients who were hospitalized diagnosed with EC based on abdominal CT findings at Kyung Hee University Hospital at Gangdong, Seoul Medical Center and Kyung Hee University Medical Center between 2006 and 2020. The inclusion criteria for the EC group were patients over 20 years of age who were admitted with dysuria, hematuria, and pyuria in whom urine examination was performed and gas formation around the bladder was confirmed by CT (Figure 1). Patients with bladder gas with urethral catheter insertion, after cystoscopy procedure, or patients with gas due to bladder damage, were excluded. Acute cystitis is diagnosed when there is sudden lower urinary tract symptoms due to a urinary tract infection of the bladder. No special radiological examination is required for diagnosis. As control group, the AC group comprised patients who were hospitalized for treatment of AC with severe symptoms including intolerable abdominal pain or gross hematuria in same period. Abdominal CT scan was performed for all patients in AC group to differentiate from other disease related to these symptoms. In both the EC and control (AC) groups, patients <20 years of age, or who were hospitalized for the treatment of other diseases, were excluded. The institutional review board of our institute approved our study and confirmed the informed consent.

### 2.3. Risk Factors Analysis

We sought to identify the clinical features and predisposing diseases such as age, sex, diabetes mellitus (DM), hypertension (HTN), cerebrovascular accident (CVA), chronic kidney disease (CKD), neurogenic bladder (NB), history of urinary tract infection (UTI), and EPN associated with the development of EC. Species identified in urine culture, sepsis, and mortality rates were also compared between the AC and EC groups.

### 2.4. Statistical Analysis

Data were analyzed using SPSS Statistics for Windows, version 17.0 (IBM SPSS, Armonk, NY, USA). Proportion comparisons for categorical variables were performed using Pearson’s Chi-squared, and Fisher’s exact. *p* values and relative ratios were calculated for the risk factors. *p* values < 0.05 were considered significant. In addition, multivariate model analysis was performed to determine the significance of EC and AC.

## 3. Results

### 3.1. Patient General Data

A total of 54 EC patients were identified, and all 92 patients with AC were set as the control group. The median (interquartile range (IQR)) age of patients with EC was older than that of the patients with AC (78.5 (15.3) years (range: 52–100) vs. 70.0 (26.5) years (range: 28–97 years)). Although the proportion of women was high in both the AC and EC groups (73.9% vs. 63.0%, respectively), the difference was particularly noticeable in the AC group. Five underlying diseases, including HTN, DM, CVA, CKD and NB, were present at a low proportion in the AC patients but prevalent in the EC patients, particularly DM (AC 15.2%, EC 63.0%) and HTN (AC 39.1%, EC 61.1%). UTI history showed similar rates in both the AC and EC groups (18.9% vs. 16.7%). No patients with EPN were detected in the AC group, whereas 10.2% of EC patients were diagnosed with EPN. In the urine culture tests, *Escherichia coli* was the most common causative agent in both the AC and EC groups (52.8% vs. 40.4%, respectively), followed by *Klebsiella pneumoniae*. Extended-spectrum beta-lactamase (ESBL)-producing bacteria were more frequently identified in the EC patients (36.5%). In the AC group, the incidence and mortality of sepsis were both zero, whereas among the 54 EC patients, 26 (48.1%) were diagnosed with sepsis and 6 died (11.1%) (Table 1).

### 3.2. Univariate and Multivariate Analysis of Risk Factors

The univariate analysis of the underlying diseases showed that sex and previous UTI were not significant (odds ratio (OR) 0.600; 95% confidence interval (CI) 0.291–1.235; *p* = 0.193 and OR 0.859; 95% CI 0.326–2.261; *p =* 0.814, respectively). However, age (OR 1.058; 95% CI 1.029–1.088, *p <* 0.001), DM (OR 9.471; 95% CI 4.286–20.930; *p <* 0.001), HTN (OR 2.444; 95% CI 1.227–4.868; *p =* 0.016), CVA (OR 3.333; 95% CI 1.454–7.641; *p =* 0.005), CKD (OR 26.471; 95% CI 5.870–119.364; *p <* 0.001), and NB (OR 8.156; 95% CI 2.742–24.264; *p <* 0.001) were significantly associated with EC. In the multivariate analysis, DM (OR 6.251; 95% CI 2.254–17.250; *p <* 0.001), CKD (OR 18.439; 95% CI 3.421–99.404; *p =* 0.001), and NB (OR 7.374; 95% CI 1.993–27.285; *p =* 0.003) were significantly associated with EC (Table 2).

## 4. Discussion

This study collected information on EC patients from three institutions and analyzed their demographic data, underlying diseases, history, and causative bacteria, and compared them to those of the AC control group to identify the risk factors that discriminated EC with symptoms similar to those of AC. As the number of patients diagnosed with EC is small, the number of related studies is correspondingly small worldwide and retrospective risk factor analysis studies are also scarce.

The 2021 European Association of Urology guideline on urological infections recommends that the diagnosis of uncomplicated cystitis is first based on the history of lower urinary tract symptoms, urinalysis and urine culture are performed, and antimicrobials therapy is applied. However, no detailed information on emphysematous cystitis or differentiation points from acute cystitis are specified.

Thomas et al. studied 135 EC patients reported in the literature between 1956 and 2006. They reported an average patient age of 61.9 years and that 64% of patients were female and 66.7% had DM. The overall mortality rate was 7% and the major pathogens were *Escherichia coli* (58%) and *Klebsiella pneumoniae* (21%). The authors emphasized that if risk factors such as DM and complicated UTI are present and there is no response to standard treatment, EC should be suspected and a CT scan is recommended for the assessment of the disease severity and extent [7].

Schicho et al. reported a mortality rate of 7.4% for EC in a total of 136 patients between 2007 and 2016. The average patient age was 67.9 ± 14.2 years. In 21 patients (15.4%), emphysematous infections of other organs were simultaneously detected, most commonly EPN. The primary pathogen identified was *Escherichia coli* (54.4%). The patients were treated primarily with conservative management, including antibiotics (*n* = 105; 77.2%). Ten of the 136 patients with EC died, corresponding to a mortality rate of 7.4%. Thus, despite the relatively low mortality rate of the EC compared to the EP, a high level of suspicion should be maintained to promote successful and conservative management [4].

Similar to the previously described studies, the incidence of sepsis in this study was 48.1% and the mortality rate was as high as 11.1%. CT is an essential test for EC as its mortality rate differs from that of general AC; thus, it is important to identify the risk factors. Several studies have identified DM as a significant risk factor [2,4,7,8,11]. In our study, in addition to DM, the significant risk factors also included CKD and NB.

Toyota et al. reported that two-thirds of the EC patients had DM compared to 10% of the general population. The high concentration of sugar in the tissue creates a favorable environment for microorganisms [12]. The fermentation of bacteria leading to high blood sugar levels mainly produces carbon dioxide, which induces gas in the bladder [13,14]. Gases generated rapidly in the tissues cannot move and accumulate, creating bubbles. Yang et al. reported tissue rupture and bleeding when the surrounding tissue was weak. In this context, the effects of DM in patients with UTIs can lead to diabetic microangiopathy and immune system impairment, in addition to bacterial growth in hyperglycemic environments. DM-induced microangiopathy impairs bladder function, leading to another EC risk factors [15]. Successful glycemic control may reduce microvascular complications by 50–60%, thereby reducing the risk of EC development [16]. Moreover, patients with uncontrolled DM show significantly reduced bactericidal activity against intracellular pathogens, which weakens host defense mechanisms, making them susceptible to infection [17].

EC patients present symptoms ranging from asymptomatic to abdominal and urinary pain. In particular, CKD patients with decreased renal function may be asymptomatic. Yokoo et al. reported an EC complication in a patient undergoing hemodialysis and suggested that, as patients with a long history of dialysis often complains of asymptomatic hematuria, careful observation and examination are necessary [18]. In 2018, Wang et al. reported a case of a 38-year-old male patient with DM, CKD, and NB as underlying diseases in which CKD was proposed to have led to urinary tract susceptibility to infection due to the decreased urinary tract irrigation effect owing to CKD. In addition, as DM and CKD coexisted, a UTI may have occurred due to decreased patient immunity [19].

Quint et al. published two EC-related case reports suggesting the high probability of developing EC in patients with underlying diabetes, neurogenic bladder, and chronic UTI. They also suggested the need to address the underlying diseases to ensure treatment success [20]. In NB, urination is not smooth and bladder retention occurs, which increases the risk of infection in the bladder. Yoshida et al. also proposed NB as a risk factor for EC, proposing that successful treatment required continuous bladder drainage and prevention of bladder retention using a urethral catheter [8].

Our study has several limitations. Since this research is a retrospective investigation, the study on factors that can influence the development of EC other than the previously identified variables is unfeasible. The control (AC) group included only hospitalized patients. The symptoms of AC are not generally serious; thus, outpatient management is typical and cases requiring hospitalization often include gross hematuria or complaints of severe discomfort. Also, physicians from different institutions may have different hospitalization criteria for cystitis patients. Thus, selective bias may have occurred in the selection of the control group. Another drawback is that laboratory findings, such as white blood cells, hemoglobins and C-reactive protein (CRP), were not analyzed in this study. Significant results may be obtained by comparing the blood and urine exams between the two groups. Later, prospective, and large-scale studies are needed to address this shortcoming.

## 5. Conclusions

The comparisons of EC and AC in this study identified DM, CKD, and NB as risk factors for EC. Thus, the treatment of general cystitis in patients with these risk factors requires active evaluations such as CT scans and suspicion of EC to prevent the development of serious conditions such as sepsis.

## Figures and Tables

**Table 1:** Baseline characteristics of patients with EC and AC.

		AC (*n* = 92)	EC (*n* = 54)	*p*-Value
Age		70.5 (26.5)	78.5 (15.3)	<0.001
Gender				0.193
	Male (*n* = 44)	24	20	
	Female (*n* = 102)	68	34	
Underlying disease				
	Diabetes Mellitus	14 (15.2)	34 (63.0)	<0.001
	Hypertension	36 (39.1)	33 (61.1)	0.016
	Cerebrovascular accident	12 (13.0)	18 (33.3)	0.005
	Chronic kidney disease	2 (2.17)	20 (37.0)	<0.001
	Neurogenic bladder	5 (5.43)	15 (31.9)	<0.001
HbA1c		6.80 ± 0.774	7.92 ± 1.89	<0.001
previous UTI history		17 (18.9)	7 (16.7)	0.814
Emphysematous Nephritis		0(0)	5 (10.2)	0.004
Urine culture				
	*Enteroccocus coli*	47 (52.8)	21 (40.4)	
	*Klebsiella pneumoniae*	2 (2.25)	18 (34.6)	
	*Enterococcus*	3 (3.37)	0 (0)	
	etc.	0 (0)	12 (23.1)	
ESBL positive		17 (17.6)	19 (36.5)	0.015
Sepsis		0 (0)	26 (48.1)	<0.001
Mortality		0 (0)	6 (11.1)	0.002

Values are presented as median (interquartile range) or mean (±range) or number (%); *p* < 0.05, statistically significant; EC: emphysematous cystitis; AC: acute cystitis; UTI: urinary tract infection; ESBL: extended-spectrum beta-lactamase.

**Table 2:** Univariate and multivariate analysis of the risk factors for emphysematous cystitis.

	Univariate Analysis		Multivariate Analysis	
	OR (95% CI)	*p*-Value	OR (95% CI)	*p*-Value
Sex	0.600 (0.291–1.235)	0.193	-	-
Age	1.058 (1.029–1.088)	<0.001	1.033 (0.994–1.074)	0.100
Diabetes mellitus	9.471 (4.286–20.930)	<0.001	6.251 (2.265–17.250)	<0.001
Hypertension	2.444 (1.227–4.868)	0.016	0.702 (0.246–1.999)	0.507
Cerebrovascular accident	3.333 (1.454–7.641)	0.005	1.684 (0.542–5.233)	0.367
Chronic kidney disease	26.471 (5.870–119.364)	<0.001	18.439 (3.421–99.404)	0.001
Neurogenic bladder	8.156 (2.742–24.264)	<0.001	7.374 (1.993–27.285)	0.003
Previous UTI history	0.859 (0.326–2.261)	0.814	-	-

*p* < 0.05, statistically significant; OR: odds ratio; CI: confidence interval; UTI: urinary tract infection; -: not applicable

## Data Availability

The data presented in this study are available on request from the corresponding author. The data are not publicly available due to privacy restrictions.

## Abbreviations

AC	acute cystitis
CKD	chronic kidney disease
CT	computed tomography
CVA	cerebrovascular accident
DM	diabetes mellitus
EC	emphysematous cystitis
EPN	emphysematous pyelonephritis
ESBL	extended-spectrum beta-lactamase
HTN	hypertension
NB	neurogenic bladder
UTI	urinary tract infection
